# Comprehensive omics‐based classification system in adult patients with B‐cell acute lymphoblastic leukemia

**DOI:** 10.1002/1878-0261.70053

**Published:** 2025-05-19

**Authors:** Yang Song, Ting Liu, Qishan Hao, Qiuyun Fang, Xiaoyuan Gong, Yan Li, Zheng Tian, Hui Wei, Min Wang, Jianxiang Wang, Tao Cheng, Yingchang Mi

**Affiliations:** ^1^ State Key Laboratory of Experimental Hematology, National Clinical Research Center for Blood Diseases, Haihe Laboratory of Cell Ecosystem, Institute of Hematology & Blood Diseases Hospital Chinese Academy of Medical Sciences & Peking Union Medical College Tianjin China; ^2^ Tianjin Institutes of Health Science Tianjin China

**Keywords:** B‐cell acute lymphocytic leukemia, hematopoietic stem cell transplantation, molecular classification, multi‐omics, tumor immune environment

## Abstract

B‐cell acute lymphoblastic leukemia (B‐ALL) is a highly heterogeneous disease with a challenging prognosis, particularly in adult patients. We enrolled 88 adult B‐ALL patients with transcriptomic and mutation profiles for classification system identification, and a comprehensive system for B‐ALL patients (COMBAT) was developed. COMBAT stratified patients into three cohorts: (1) COMBAT1, characterized by high stem/myeloid antigen expression, low immune infiltration, high infiltration of endothelial cells, and hypo‐CIMP (CpG island methylator phenotype); (2) COMBAT2, defined as an inflamed subtype with immune exhaustion, moderate myeloid antigen expression, and hypo‐CIMP; and (3) COMBAT3, marked by proliferative profiles with MYC pathway activation and hypomethylation at enhancer regions in patients characterized by CIMP. The molecular features of the three COMBATs were verified in two external cohorts, the GSE34861 (*N* = 194) and GSE66005 (*N* = 109) datasets. In univariate analysis, only COMBAT classification presented significance for OS, and patients of COMBAT3 presented significantly superior survival than COMBAT1/2 in Ph‐negative ALL. Ph‐negative ALL patients undergoing allogeneic hematopoietic stem cell transplantation (allo‐HSCT) in the COMBAT3 group showed better overall survival (OS) than those in the COMBAT1‐2 groups (estimated 3‐year OS: 100% vs. 65.6%, *P* = 0.034), suggesting a prognostic benefit of this subtype. In summary, the COMBAT system redefines the characteristics of adult B‐ALL subtypes and guides the selection of allo‐HSCT for Ph‐negative patients.

## Graphical abstract

The COMBAT classification system, developed through multi‐omics integration, stratifies adult patients with B‐cell acute lymphoblastic leukemia(B‐ALL) into three molecular subtypes with distinct surface antigen patterns, immune landscape, methylation patterns, biological pathways and prognosis. COMBAT3 shows superior survival, particularly in Philadelphia chromosome (Ph)‐negative patients post allogeneic hematopoietic stem cell transplantation (allo‐HSCT). This framework enables precision prognosis and guides therapeutic stratification.

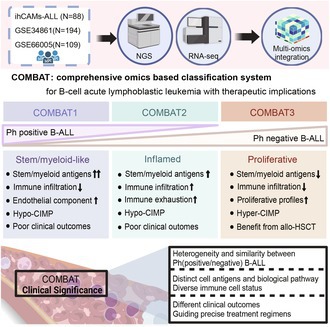

**Abbreviations**
allo‐HSCT: allogeneic hematopoietic stem cell transplantation
B‐ALL: B‐cell acute lymphocytic leukemia
BMNCs: bone marrow mononuclear cells
CIMP: CpG island methylator phenotype
COMBAT: comprehensive omics‐based classification system for B‐cell acute lymphoblastic leukemia with therapeutic implications
CR: complete remission
DEG: differentially expressed gene
EFS: event‐free survival
MRD: minimal residual disease
ND: newly diagnosed
NGS: next‐generation sequencing
NTP: nearest template prediction
OS: overall survival
Ph: Philadelphia chromosome
RR: refractory/relapsed
SES: stromal enrichment score
TME: tumor microenvironment
WBC: white blood cell

## 1 Introduction

B‐cell acute lymphocytic leukemia (B‐ALL) is a hematopoietic malignancy characterized by abnormal differentiation and proliferation of malignant lymphoid precursors [1]. Although the cure rate of pediatric ALL is >90% [2], it remains a devastating disease in older adolescents (14–18 years old) and adults [3], which indicates the difference of leukemogenic pathways between children and adults.

The last decades have witnessed great progress in the treatment options for adults with B‐ALL. First, the pediatric‐inspired dose‐intensive regimen greatly improved outcomes in adult B‐ALL patients, with a complete remission (CR) rate of 90+% and a 5‐year overall survival (OS) rate of 40% [4, 5]. The incorporation of BCR‐ABL1 tyrosine kinase inhibitors into conventional chemotherapy has markedly improved outcomes for patients with Philadelphia chromosome (Ph)‐positive ALL [6].

In Ph‐negative ALL, the integration of blinatumomab and inotuzumab into frontline therapy has improved the estimated 3‐year survival rate to 80% [4]. Additionally, CAR‐T cell therapies have shown significant efficacy in refractory/relapsed (RR) patients [7, 8, 9]. Despite the promise of emerging immune‐targeted drugs, the availability of blinatumomab and inotuzumab is limited worldwide, especially in developing countries, and the availability of CAR‐T cells is even more limited. Intensive chemotherapy remains the frontline therapy for most ALL patients in China, and allogeneic hematopoietic stem cell transplantation (allo‐HSCT) remains the cornerstone of treatment for ALL [10].

B‐ALL is a highly heterogeneous disease, which consists of dozens of subtypes with distinct cytogenetic alterations and molecular abnormalities. Although tools like ALLSpice [11], ALLSorts [12], ALLCatchR [13], and MD‐ALL [14] rely on transcriptomic data for B‐ALL subtyping, these platforms primarily focus on B‐ALL diagnosing classification and risk stratification, with most designed for pediatric patients or datasets spanning both pediatric and adult cases. Given the differences in molecular composition and prognoses between pediatric and adult B‐ALL, an optimal method specifically for adult subtype classification remains undefined. Furthermore, molecular subtyping using multi‐omics data must account for numerous prognostic factors, such as age, white blood cell (WBC) counts, and minimal residual disease (MRD), which significantly influence patient outcomes. However, a comprehensive classification system that integrates diverse subtypes and provides greater clinical relevance with clear prognostic and therapeutic implications in B‐ALL is still lacking.

In our study, we introduce a comprehensive omics‐based classification system for B‐cell acute lymphoblastic leukemia with therapeutic implications (COMBAT) based on our cohort of older adolescent and adult patients via 10 clustering algorithms. This novel approach offers a more comprehensive understanding of molecular features, enhances survival prediction, and aids in determining the suitability of transplantation for patients.

## 2 Methods

### 2.1 Participants in the ihCAMs‐ALL cohort

This retrospective study was conducted from June 2020 to June 2024 at the Institute of Hematology and Blood Diseases Hospital in Tianjin, China. The cohort titled “ihCAMs‐B‐ALL” comprised 69 newly diagnosed (ND) and 19 RR B‐ALL patients. The cohort included 43 males (49%) and 45 females (51%) with a median age of 34 years (range: 15–70). The study was approved by the Hospital Ethics Committee and conducted in accordance with the Declaration of Helsinki (Ethical batch number: KT2020004‐EC‐2, NSFC 2022038‐EC‐2). Written informed consent was obtained from all patients. All B‐ALL patients were treated according to the IH‐2014 protocol as described by Gong *et al*. [15], and imatinib or dasatinib was added to the treatment for Ph‐positive patients from day 8 of induction chemotherapy. Among the patients in the ND group, 38 underwent allo‐HSCT. The median follow‐up time for the study cohort was 28 (5–39) months.

### 2.2 RNA sequencing, next‐generation sequencing, and methylation 850 K profiling on ihCAMs‐B‐ALL cohort

All participants (*N* = 88) in the ihCAMs‐B‐ALL cohort along with 9 healthy donors underwent RNA‐seq. Next‐generation sequencing (NGS) was performed on all “ihCAMs‐B‐ALL” cases using a customized panel targeting 267 hotspot mutations associated with malignant hematologic disorders (Table S1). DNA methylation was conducted on bone marrow mononuclear cells (BMNCs) from 34 Ph‐negative patients. Among the ND B‐ALL patients, 42% were classified as Ph‐positive B‐ALL, 51% as Ph‐negative B‐ALL, and 7% as Ph‐like B‐ALL.

RNA sequencing (RNA‐seq): Total RNA was isolated from frozen BMNCs using the RNA Assay Kit in combination with the Qubit® 2.0 Fluorometer system (Life Technologies, CA, USA). Samples with RIN values exceeding 7.0 were selected for downstream processing. Library preparation was conducted following the protocol of the NEBNext® Ultra™ RNA Library Prep Kit (New England Biolabs, USA). mRNA was selectively enriched, fragmented, and converted into cDNA. The resulting cDNA libraries underwent end‐repair, adapter ligation, and PCR amplification. Final libraries were sequenced on the Illumina NovaSeq 6000, generating 150 bp paired‐end reads.

Paired‐end reads were aligned to the GRCh38 human genome reference using HISAT2 v2.1.0. Gene annotation files, obtained from Ensembl (http://www.ensembl.org/), were employed for STAR‐based mapping and subsequent evaluation of gene expression levels. Fragment counts per kilobase of non‐overlapping exon per million mapped fragments were initially computed and then converted into transcripts per kilobase million (TPM) values.

Next‐generation sequencing (NGS): NGS was performed on the Illumina NovaSeq 6000 platform. Raw FASTQ files were processed using fastp (v0.23.2) [16] to remove adapters and low‐quality reads. Processed reads were aligned to the reference genome, and mutations were called by the Dragen pipeline (v3.10.4). Candidate somatic mutations with a variant allele frequency (VAF) >0.5% were selected, manually reviewed, and validated using the Integrative Genomics Viewer (IGV).

Methylation 850 K: Genomic DNA was extracted from BMNCs using the DNeasy Blood and Tissue Kit (Qiagen). DNA purity and concentration were assessed via Nanodrop 2000 (ThermoScientific). Bisulfite treatment was performed with the EZ DNA Methylation Gold Kit (Zymo Research, USA), and the resulting DNA was hybridized to the Illumina Infinium MethylationEPIC BeadChip array.

Raw signal intensities were extracted from IDAT files using the R package ‘ChAMP’ [17], which also calculated β values for each probe as M/(M + U), where M and U represent the mean methylated and unmethylated signal intensities, respectively. Comprehensive filtering criteria were applied, including the removal of probes with detection *P*‐values >0.01, probes with fewer than three beads in at least 5% of samples, non‐CpG probes, SNP‐related probes, multi‐hit probes, and probes on sex chromosomes (X and Y) [18]. Differentially methylated probes (DMPs) were identified using default settings. To define the CpG island methylator phenotype (CIMP) in B‐ALL, probes in CpG islands with β values >0.9 in normal samples were excluded to minimize background noise, and only probes with high variability (standard deviation >0.25) across ALL samples were analyzed [19].

### 2.3 Flow cytometry analysis

Flow cytometry analyses were performed on diagnostic BM aspirates identified by CD45 versus light side‐scatter properties. A screening panel of four tubes was conducted: (1) CD64/CD117/CD34/CD33/CD7/HLA‐DR/CD38/CD45; (2) CD15/CD13/CD34/CD123/CD56/CD16/CD11b/CD45; (3) CD36/CD10/CD5/CD20/CD4/CD14/CD19/CD45; (4) TdT/MPO/CD9/CD2/cCD79a/mCD3/cCD3/CD45.

Surface antigen expression was categorized into three levels: negative, low expression positive signal, and high expression positive signal. Classification was determined based on flow cytometry using negative and positive controls. Antigen expression was defined as negative if the blasts did not express the marker when compared with a population expected to express the marker. Expression was classified as positive if the blasts expressed the marker relative to a population not expected to express it. If the expression level fell between these two extremes, it was defined as weak positive. Partially positive cases were defined as 20%–75% of blasts showing expression. Both weak positive and partially positive cases were grouped under low expression positive signals, while positive cases were classified as high expression positive signals.

MRD was measured using BM aspirates on post‐induction (MRD1, days 29–42) and post the first consolidation (MRD2). MRD levels of <0.01% and ≥0.01% were considered as negative and positive, respectively.

### 2.4 Multi‐omics bioinformatics analysis

For RNA‐seq data, differentially expressed genes (DEGs) among different groups were calculated using the “limma” package [20] with significance thresholds of both |log2FoldChange| ≥1 and adjusted *P* < 0.05. For GSEA based on RNA‐seq data, we prepared a preranked gene list according to the descending‐ordered log2FoldChange value derived from differential expression analysis; we then leveraged the R package “clusterprofiler” [21] to determine the functional enrichment based on the Hallmark pathway [22].

Mutation signatures were used to decipher samples that shared similar mutational spectra [23]. All non‐single‐base substitutions (e.g., insertions, deletions, and complex multibase substitutions) were filtered out, leaving single‐base substitution mutations annotated as nonsense, missense, or coding silent substitutions [24]. The mutational signatures curated at http://cancer.sanger.ac.uk/cosmic/signatures were evaluated via the R package deconstructsigs (v1.9.0) with the following parameters: “exome2genome” trinucleotide‐count normalization [25].

### 2.5 Multi‐omics consensus ensemble by integrative clustering

In our study, we categorized B‐ALL patients primarily based on an integrated analysis of transcriptomics and somatic mutation data. This classification was accomplished using the widely recognized 10 unsupervised integrative clustering methods, implemented through the MOVICS R package [26, 27], particularly employing the “getConsensusMOVICS()” function. We employed the clustering prediction index (CPI) and Gap statistics to determine the optimal number of subtypes. Additionally, we used silhouette scores as the key metric to evaluate the similarity in sample clustering, ensuring robust classification. To ensure comprehensive coverage, our feature selection included transcriptomic mRNAs and lncRNAs expressions and somatic mutations, thereby providing a broad and informative dataset for our clustering algorithms. To better fit the model and accelerate the clustering efficiency, features with flat values were removed. Specifically, we selected the top 1000 most variable mRNAs and lncRNAs according to the median absolute deviation. Additionally, genes with mutation rates >3% (*N* = 39) were selected for subtyping [28].

### 2.6 Estimation of B‐ALL microenvironment cell abundance

To assess the tumor microenvironment (TME) composition of each ALL case, we used the R package MCPcounter [29], which provides abundance scores for eight immune populations (T cells, CD8+ T cells, cytotoxic lymphocytes, natural killer cells, B‐cell lineage, monocytic lineage, myeloid dendritic cells, and neutrophils) and two stromal populations (endothelial cells and fibroblasts). To determine the functional orientation of TME, we used signatures derived from the literature including immunosuppression (*CXCL12*, *TGFB1*, *TGFB3*, and *LGALS1*), T‐cell activation (*CXCL9*, *CXCL10*, *CXCL16*, *IFNG*, and *IL15*), T‐cell survival (*CD70* and *CD27*), Tregs (*FOXP3* and *TNFRSF18*), major histocompatibility complex class I (*HLA‐A*, *HLA‐B*, *HLA‐C*, *HLA‐E*, *HLA‐F*, *HLA‐G*, and *B2M*), myeloid cell chemotaxis (*CCL2*), and tertiary lymphoid structures (TLSs) (*CXCL13*). We also collected four immune suppression‐associated signatures including tumor‐infiltrating Tregs, myeloid‐derived suppressor cell (MDSC), C‐ECM, and Wnt/TGF‐β [30]. Scores for each signature were calculated as the geometric mean of signature expression. The presence of infiltrating immune/stromal cells in the tumor tissue was estimated using the R package “estimate” [31]. The methods mentioned above have also been described in an earlier study [19].

### 2.7 External validation cohort for validation

To validate the COMBAT findings, we sourced additional adult B‐ALL cohorts with detailed clinical data and gene expression data from the Gene Expression Omnibus (GEO) database, including GSE34861 (*N* = 194) and GSE66005 (*N* = 109). Transcriptomic data were standardized using log2‐transformed TPM. To minimize batch effects, we applied the “ComBat” algorithm from the “sva” R package (version 3.29.1) [32].

In addition, 8 B‐ALL cell lines from the Cancer Cell Line Encyclopedia (CCLE) databases with available RNA‐seq data were used. Each sample in the external cohort was further classified into COMBAT subtypes using the nearest template prediction (NTP) algorithm [33]. For NTP prediction, we utilized normalized gene expression data from RNA‐seq, applying a log2 transformation [log2(TPM + 1)] to ensure comparability across samples.

### 2.8 Statistical analysis

All the statistical analyses were implemented using R software (version 4.2.2) or GraphPad Prism software (version 9.5.0). For survival analysis, Overall survival (OS) was defined as the time from treatment initiation to death or last follow‐up. Event‐free survival (EFS) was defined as the time from treatment initiation until assessment of response after the first cycle of chemotherapy; if the participant failed to achieve CR, the date of relapse or death or last follow‐up in those achieving CR was considered. Survival analyses were estimated using the Kaplan–Meier method and compared using the log‐rank test. Descriptive statistics were assessed using Fisher's exact test for categorical data, and comparisons of continuous variables were performed using the Kruskal–Wallis test as appropriate. Univariate and multivariate analyses were carried out by Cox proportional hazards regression for OS and EFS. A *P*‐value of less than 0.05 was considered statistically significant in all unadjusted analyses. To control the false discovery rate (FDR), adjusted *P*‐values were calculated using the Benjamini‐Hochberg method.

## 3 Results

### 3.1 Transcriptional and immune characteristics of B‐ALL

**Fig. 1:**
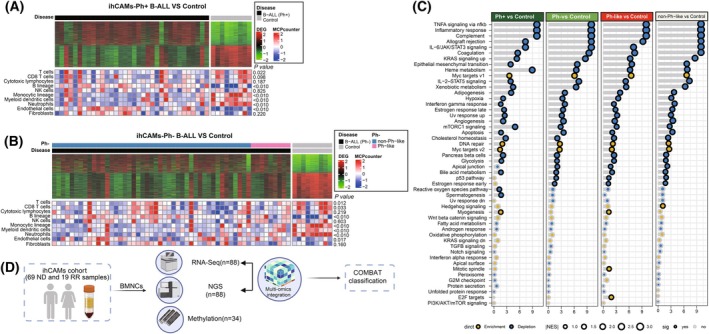
Omics comparison of adult B‐cell acute lymphoblastic leukemia (B‐ALL) in ihCAMs‐B‐ALL cohort. (A, B) The heatmap depicted the transcriptomics and immunocyte infiltration profiles in Philadelphia chromosome (Ph)‐positive B‐ALL (A) and Ph‐negative B‐ALL (B) patients from the ihCAMs cohort, each compared with healthy controls. In the context, Ph‐negative B‐ALL includes Ph‐like B‐ALL and non‐Ph‐like B‐ALL. ihCAMs, name of cohort derived from our center. (C) The gene set enrichment analysis (GSEA) dot plot for hallmark pathways illustrating the enriched pathways in Ph‐positive, Ph‐negative, Ph‐like, and Ph‐negative B‐ALL patients excluding Ph‐like patients(non‐Ph‐like) compared with healthy controls, respectively. The blue dots indicate downregulated pathways, while the yellow dots indicate upregulated pathways. The size of the dots represents the |NES| (Normalized Enrichment Score) value, with larger dots corresponding to higher |NES| values. Statistical significance was determined using Fisher's exact test. The dots with black outlines indicate statistical significance, while those with gray outlines denote no statistical significance. (D) The sequencing design performed on the ihCAMs cohort. BMNCs, bone marrow mononuclear cells; ND, newly diagnosed; NGS, next‐generation sequencing; RR, refractory/relapsed.

The transcriptomic profiles of B‐ALL patients differed significantly from those of healthy controls. When comparing with healthy controls, we observed the upregulation of 2230 genes and the downregulation of 1116 genes in Ph‐positive patients (Fig. 1A). Similarly, Ph‐negative patients presented the upregulation of 2240 genes and the downregulation of 1195 genes (Fig. 1B).

The analysis of immune infiltration status compared with that in healthy controls revealed an immunocompromised state in both Ph‐positive and Ph‐negative patients, which was characterized by the downregulation of T cells and myeloid‐derived cells, including monocytes, myeloid dendritic cells, and neutrophils. Conversely, endothelial cell infiltration levels were greater in B‐ALL patients than in the control group (Fig. 1A,B).

Compared to healthy controls, B‐ALL patients exhibited significant activation of the MYC target pathway and DNA repair pathway (Fig. 1C). Intriguingly, we noted that immune‐associated pathways were downregulated in all B‐ALL subtypes compared with healthy controls, which was consistent with the reduced immunocyte infiltration observed above.

### 3.2 Multi‐omics ensemble identifies three COMBAT phenotypes

**Fig. 2:**
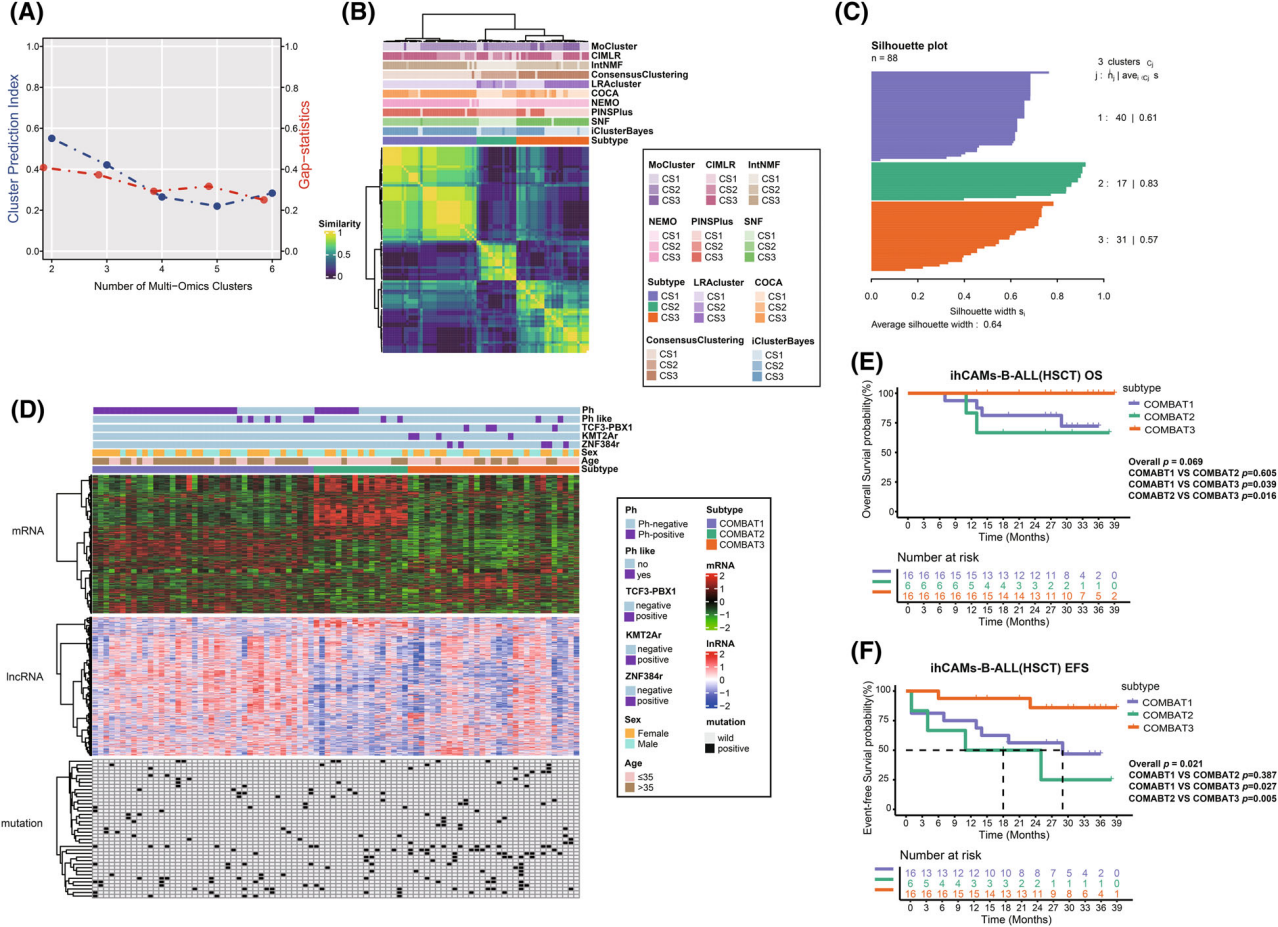
Identification of three distinct COMBAT clusters and evaluation of diverse clinical outcomes. (A) Identification of optimal cluster number by calculating Consensus Partitioning Index (CPI) and Gap statistics in ihCAMs cohort. CPI is depicted by the blue line, where a higher CPI value signifies enhanced stability and reliability of the identified clusters. The red line represents the gap statistic, which determines the optimal number of clusters by contrasting the change in within‐cluster dispersion with an expected null reference data distribution. A peak in the gap statistics indicates the most appropriate number of clusters. (B) Consensus Matrix Similarity Heatmap Utilizing 10 Clustering Algorithms: This heatmap represents the consensus matrix similarity, derived from the application of 10 commonly used clustering algorithms using the R package “MOVICS”. CS1: Cluster 1; CS2: Cluster 2; CS3: Cluster 3. (C) Silhouette Plot for Consensus Clustering shows the consensus clustering effect of the three subtypes, with an average silhouette score of 0.64. (D) The consensus heatmap displays transcriptomic profiling, including mRNA and lncRNA expression and genetic mutations, organized from top to bottom. KMT2Ar, KMT2A rearrangement; ZNF384r, ZNF384 rearrangement. (E) Kaplan–Meier curve of overall survival (OS) for all patients undergoing allogeneic hematopoietic stem cell transplantation (allo‐HSCT) across the three molecular subtypes in ihCAMs cohort. Overall *P*, Fisher's exact test; pairwise *P*, Log‐Rank test. (F) Kaplan–Meier curve of event‐free survival (EFS) for all patients undergoing allo‐HSCT across the three molecular subtypes in ihCAMs cohort. Overall *P*, Fisher's exact test; pairwise *P*, Log‐Rank test.

To further understand the heterogeneity of B‐ALL, we enrolled transcriptomic and gene mutation data of 88 B‐ALL patients from the ihCAMs‐B‐ALL cohort for consensus multi‐omics analysis (Fig. 1D). Based on CPI, Gaps‐statistics analysis, and clinical relevance, we determined that the number of 3 was a reasonable choice for B‐ALL subtyping (Fig. 2A). Subsequently, the integration of the clustering results derived from the 10 algorithms was performed via consensus ensembles. To make the classification more robust, patients were divided into three clusters (Fig. 2B). The silhouette scores for the three subtypes were 0.61, 0.83, and 0.57, respectively (Fig. 2C), indicating high stability within each cluster. Accordingly, the results demonstrated that the clusters were well apart from each other, and three robust COMBAT subtypes were clearly distinguished (Fig. 2D).

Within the ihCAMs‐B‐ALL cohort, Ph‐positive patients were categorized into two clusters (COMBAT1‐2), whereas Ph‐negative patients were segmented into three clusters (COMBAT1‐3). Notably, 80% (8/10) of Ph‐like patients were identified in COMBAT1 and COMBAT2 (Table 1). We also conducted principal component analysis (PCA) between Ph‐positive and Ph‐negative patients (Fig. S1A), as well as ND and RR patients across the three subtypes (Fig. S1B). The results revealed that common features were present within the same subtype, not only between Ph‐positive and Ph‐negative patients, but also between ND and RR patients. Additionally, a previous study has demonstrated that the transcriptomic phenotypes of RR and ND BCR‐ABL positive B‐ALL are largely similar [34], which further supports the stability of our molecular subtyping. Consequently, we included both Ph‐positive and Ph‐negative patients, as well as ND and RR patients, in our COMBAT consensus ensembles. In addition, the COMBAT2 group of Ph‐negative patients contained a higher proportion of RR patients in comparison to the COMBAT1 and COMBAT3 groups (21.4% vs. 66.7% vs. 16.1%, *P* = 0.0088, Table 1).

**Table 1:** Clinical characteristics in ihCAMs‐B‐ALL cohort under COMBAT classification. Allo‐HSCT, allogeneic hematopoietic stem cell transplantation; CR, complete remission; KMT2Ar, KMT2A rearrangement; ND, newly diagnosed; NCCN, national comprehensive cancer network; Ph+, Philadelphia chromosome (Ph)‐positive; Ph−, Ph‐negative; RR, refractory/relapsed; ZNF384r, ZNF384 rearrangement.

	Entire cohort							
	Total	Ph + (*N* = 34)			Ph‐(*N* = 54)			
	88	COMBAT1 (*N* = 26)	COMBAT2 (*N* = 8)	*P* value	COMBAT1 (*N* = 14)	COMBAT2 (*N* = 9)	COMBAT3 (*N* = 31)	*P* value
Disease status				0.3472				0.0088
ND	69 (78.4%)	23 (88.5%)	6 (75.0%)		11 (78.6%)	3 (33.3%)	26 (83.9%)	
RR	19 (21.6%)	3 (11.5%)	2 (25.0%)		3 (21.4%)	6 (66.7%)	5 (16.1%)	
Ph‐like				–				0.0138
Yes	10 (11.4%)	0	0		6 (42.9%)	2 (22.2%)	2 (6.5%)	
No	78 (88.6%)	26 (100%)	8 (100%)		8 (57.1%)	7 (77.8%)	29 (93.5%)	

	Patients of newly diagnosed							
	Total	Ph + (*N* = 29)			Ph‐(*N* = 40)			
	69	COMBAT1 (*N* = 23)	COMBAT2 (*N* = 6)	*P* value	COMBAT1 (*N* = 11)	COMBAT2 (*N* = 3)	COMBAT3 (*N* = 26)	*P* value
Age (range)	33 (15–70)	42 (20–70)	30 (16–43)	0.0245	42 (15–67)	32 (17–35)	26.5 (15–53)	0.3231
Age range				0.0101				0.1704
<35	35 (50.7%)	6 (26.1%)	5 (83.3%)		4 (36.4%)	2 (66.7%)	18 (69.2%)	
≥35	34 (49.3%)	17 (73.9%)	1 (16.7%)		7 (63.6%)	1 (33.3%)	8 (30.8%)	
Gender				0.6302				0.7239
Female	39 (56.5%)	14 (60.9%)	3 (50.0%)		5 (45.5%)	2 (66.7%)	15 (57.7%)	
Male	30 (43.5%)	9 (39.1%)	3 (50.0%)		6 (54.5%)	1 (33.3%)	11 (42.3%)	
WBC (range, ×10^9^/L)	26.83 (1.11–261.39)	46.5 (3.69–172.92)	13.14 (2.06–30.91)	0.0368	25.78 (1.11–196.69)	8.4 (1.68–42.11)	12.58 (2.47–261.39)	0.5885
WBC range (×10^9^/L)				0.0332				0.5119
≥30	31 (44.9%)	15 (65.2%)	1 (16.7%)		5 (45.5%)	2 (66.7%)	9 (34.6%)	
<30	38 (55.1%)	8 (34.8%)	5 (83.3%)		6 (54.5%)	1 (33.3%)	17 (65.4%)	
Immunotype				0.4541				0.0346
Pro‐B	5 (7.2%)	0	0		0	0	5 (19.2%)	
Common B	54 (78.3%)	21 (91.3%)	6 (100.0%)		11 (100.0%)	3 (100.0%)	13 (50.0%)	
Pre‐B	10 (14.5%)	2 (8.7%)	0		0	0	8 (30.8%)	
Cytogenetic abnormality				–				–
ZNF384r	3 (4.3%)	0	0		0	0	3 (11.5%)	
KMT2Ar	4 (5.8%)	0	0		0	0	4 (15.4%)	
TCF3‐PBX1	3 (4.3%)	0	0		0	0	3 (11.5%)	
Ph‐like								0.0189
Yes	5 (7.2%)	0	0	–	4 (36.4%)	0	1 (3.8%)	
No	64 (92.8%)	23 (100%)	6 (100.0%)		7 (63.6%)	3 (100.0%)	25 (96.2%)	
IKZF1 gene deletion				0.1208				0.0247
Yes	26 (37.7%)	7 (30.4%)	0		6 (54.5%)	0	4 (15.4%)	
No	43 (62.3%)	16 (69.6%)	6 (100.0%)		5 (45.5%)	3 (100.0%)	22 (84.6%)	
Allo‐HSCT				0.2328				0.2522
Yes	38 (55.1%)	10 (43.5%)	4 (66.7%)		6 (54.5%)	2 (66.7%)	16 (61.5%)	
No	25 (36.2%)	12 (52.2%)	1 (16.7%)		2 (18.2%)	1 (33.3%)	9 (34.6%)	
Unknown	6 (8.7%)	1 (4.3%)	1 (16.7%)		3 (27.3%)	0	1 (3.8%)	
2024NCCN risk group				0.0006				0.11
Poor risk	52 (75.4%)	20 (87.0%)	1 (16.7%)		11 (100.0%)	2 (66.7%)	18 (69.2%)	
Standard risk	17 (24.6%)	3 (13.0%)	5 (83.3%)		0	1 (33.3%)	8 (30.8%)	

### 3.3 Patients undergoing allo‐HSCT within different subtypes experience diverse clinical outcomes

The clinical survival outcomes of ND patients within the three clusters were further investigated. Although no significant differences in survival were observed among the COMBAT clusters in the entire patient cohort (Fig. S2A,B), patients undergoing allo‐HSCT in the COMBAT3 group displayed better overall survival compared with those in the COMBAT1 and COMBAT2 groups (COMBAT1 vs. COMBAT2 vs. COMBAT3 estimated 3‐year OS rate: 72.2% vs. 66.7% vs. 100.0%, *P* = 0.069, Fig. 2E; COMBAT1 + COMBAT2 vs. COMBAT3 estimated 3‐year OS rate: 70.2% vs.100%, *P* = 0.027, Fig. S2C). Meanwhile, patients undergoing allo‐HSCT in the COMBAT3 group displayed better EFS compared with those in the COMBAT1 and COMBAT2 groups (COMBAT1 vs. COMBAT2 vs. COMBAT3 median EFS time:29 months vs. 18 months vs. not reached, *P* = 0.021, Fig. 2F; COMBAT1 + COMBAT2 vs. COMBAT3: 25 months vs. not reached, *P* = 0.033, Fig. S2D). Among the three COMBAT groups, the COMBAT2 group exhibited the poorest survival, with COMBAT1 showing intermediate prognosis and COMBAT3 showing the most favorable prognosis.

**Fig. 3:**
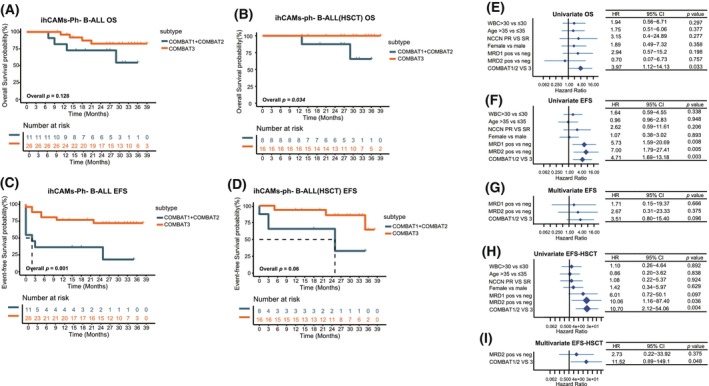
Differential survival outcomes of Philadelphia chromosome (Ph)‐negative patients of the three subtypes. (A, B) The Kaplan–Meier survival curve compares the overall survival (OS) differences among all Ph‐negative patients (A) and Ph‐negative patients undergoing allogeneic hematopoietic stem cell transplantation (allo‐HSCT, B) under the COMBAT classification. Statistical significance was determined using Log‐Rank test. (C, D) The Kaplan–Meier survival curve compares event‐free survival (EFS) differences among all Ph‐negative patients (C) and Ph‐negative patients undergoing allo‐HSCT(D)under the COMBAT classification. Statistical significance was determined using Log‐Rank test. (E–I) Univariate and multivariate cox proportional hazards model analysis of Ph‐negative patients incorporating white blood cell (WBC) count, age, national comprehensive cancer network (NCCN) risk stratification, sex, minimal residual disease (MRD), and COMBAT subtyping in relation to clinical outcomes (*N* = 40): (E) Univariate cox for OS. (F) Univariate cox analysis for EFS. (G) Multivariate cox analysis for EFS. (H) Univariate cox analysis for EFS of Ph‐negative patients undergoing allo‐HSCT. (I) Multivariate cox analysis for EFS of Ph‐negative patients undergoing allo‐HSCT.

Among Ph‐negative patients, although the COMBAT3 group did not show a significant difference compared to the COMBAT1‐2 (3‐year estimated OS rate: 82.6% vs. 54.5%, *P* = 0.128, Fig. 3A), patients who underwent allo‐HSCT in the COMBAT3 group demonstrated superior survival compared with those in the COMBAT1‐2 group (3‐year estimated OS rate: 100.0% vs. 65.6%, *P* = 0.034, Fig. 3B). Specifically, among all Ph‐negative patients, the COMBAT3 group showed superior EFS compared to COMBAT1‐2 (median EFS time of all Ph‐negative patients: not reached vs. 2.5 months, *P* = 0.001, Fig. 3C; median EFS time of Ph‐negative patients undergoing allo‐HSCT: not reached vs. 25 months, *P* = 0.06, Fig. 3D).

However, among Ph‐positive patients, there were no significant survival differences across the COMBAT clusters, regardless of allo‐HSCT status (Fig. S3A–D). These results underscore the potential prognostic benefits of the COMBAT3 classification in the context of allo‐HSCT, while patients in the COMBAT2 group appeared to face poorer outcomes even when undergoing allo‐HSCT in Ph‐negative patients.

### 3.4 Univariate and multivariate analysis of survival outcomes in Ph‐negative patients

To better determine the independent impact of different factors on survival in Ph‐negative patients, we performed univariate and multivariate analyses including these variables: WBC count, age, sex, NCCN risk group [35] (Table 1), MRD levels post‐induction (MRD1), MRD levels post the first consolidation (MRD2), and COMBAT classification. In univariate analysis, only COMBAT classification presented significance for OS, and patients of COMBAT3 presented significantly superior survival than COMBAT1/2 (Fig. 3E). MRD1‐pos, MRD2‐pos, and COMBAT1/2 predicted a worse EFS in univariate analysis (Fig. 3F,H), but significance was retained only for COMBAT1/2 in patients undergoing HSCT in multivariate analysis (Fig. 3G,I). These results highlight the improved sensitivity of COMBAT classification for predicting the survival of Ph‐negative patients, especially in those undergoing allo‐HSCT, compared with the NCCN classification or MRD in a small cohort.

### 3.5 Associations between distinct surface antigen expression patterns and biological pathways in Ph‐negative B‐ALL patients

**Fig. 4:**
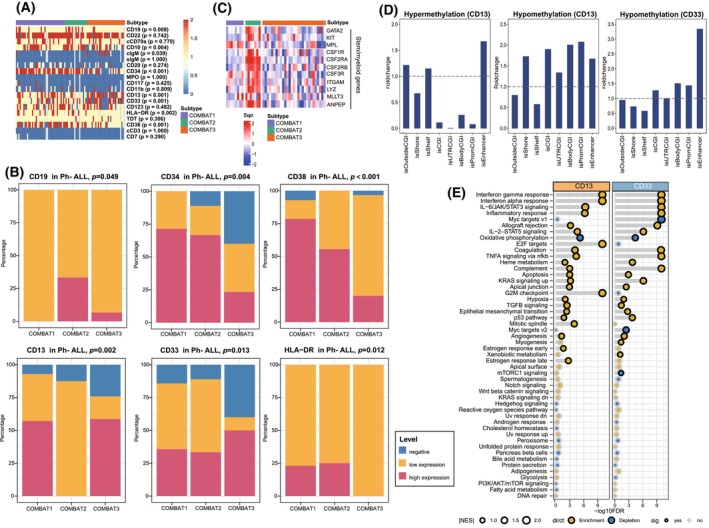
Surface antigen expression among three subtypes. (A) The heatmap depicts the expression levels of key surface antigens—including stem cell markers, myeloid lineage markers, and lymphocyte‐associated markers—across all patients classified under the COMBAT system. Antigen selection was based on established immunophenotyping criteria for acute leukemia. Statistical significance was determined using Fisher's exact test. (B) Proportions of positive blasts for selected surface antigen, including stem cell antigens and myeloid antigens among three subtypes of Philadelphia chromosome (Ph)‐negative B‐ALL patients. Statistical significance was determined using Fisher's exact test. (C) Heatmap showing expression profiles of key stemness and myeloid‐related genes across the three Ph‐negative subtypes, with genes selected according to prior research, highlighting a subset of stem and myeloid lineage genes specific to each COMBAT subtype. (D) Bar plot illustrating the region‐specific distribution of hypermethylated (left) and hypomethylated (middle) differentially methylated probes (DMPs) enriched in CD13‐positive patients, and hypermethylated DMPs (right) enriched in CD33‐positive patients, in Ph‐negative B‐ALL patients, with no enrichment of hypomethylated DMPs observed in CD33‐positive patients. (E) The gene set enrichment analysis (GSEA) dot plot for hallmark pathways illustrating the regulated pathways among CD13 and CD33 positive patients compared to those negative patients. The blue dots indicate downregulated pathways, while the yellow dots indicate upregulated pathways. The size of the dots represents the |NES| (Normalized Enrichment Score) value, with larger dots corresponding to higher |NES| values. Statistical significance was determined using Fisher exact test. The dots with black outlines indicate statistical significance, while those with gray outlines denote no statistical significance.

To understand the diverse clinical outcomes across the three clusters, we further analyzed the immunophenotype of three COMBAT subtypes through flow cytometry. Significant differences in the expression of key surface antigens, such as stem cell‐like markers (CD34, CD38, and HLA‐DR), myeloid cell‐like markers (CD13 and CD33), and B‐cell markers (CD19 and CD10), were detected among the three clusters (Fig. 4A).

Owing to the notable difference in overall survival observed in the COMBAT3 subtype, which comprises exclusively Ph‐negative patients, we carried out a detailed analysis of antigen expression, specifically concentrating on the Ph‐negative patient population. Intriguingly, patients in the COMBAT3 subgroup presented lower expression of the myeloid‐associated antigens CD13 (*P* = 0.002) and CD33 (*P* = 0.013), as well as the stem cell‐associated antigens CD34 (*P* = 0.004) and HLA‐DR (*P* = 0.012) (Fig. 4B). Additionally, stem/myeloid lineage gene expression, selected according to prior research [34], was enriched in COMBAT1‐2 (Fig. 4C), consistent with trends in surface antigen expression across the subtypes. These findings underscore the potential impact of distinct immunophenotypic profiles on patient outcomes across different subtypes.

Furthermore, we investigated differential methylation probes between Ph‐negative patients with or without the expression of myeloid markers CD13 and CD33. Differences in myeloid antigen expression are implicated in transitions at methylated CpG regions. For example, CD13‐positive patients presented hypomethylated probes across CGIs, promoter CGIs, and enhancer regions, whereas hypermethylated probes were predominantly found in enhancer regions (Fig. 4D). Hypomethylated probes were also observed across CGIs, promoter CGIs, and enhancer regions in CD33‐positive patients (Fig. 4D). These data suggested that epigenetic alterations might contribute to the differences in the expression of surface antigens among the three COMBAT groups.

To gain deeper insights into the biological processes associated with myeloid antigen expression, we conducted pathway enrichment analysis to identify significant signaling pathways (Fig. 4E). Compared with patients who were negative for these antigens, patients who were positive for CD13 and CD33 exhibited activation of immune‐associated pathways, such as Interferon‐alpha/gamma, TNF, inflammatory response, and IL6/JAK/STAT3 signaling. Additionally, cell cycle‐associated pathways, including E2F targets and the G2/M checkpoint, were significantly upregulated in CD13‐positive patients.

### 3.6 Distinct CIMPs in subsets of Ph‐negative B‐ALL patients

**Fig. 5:**
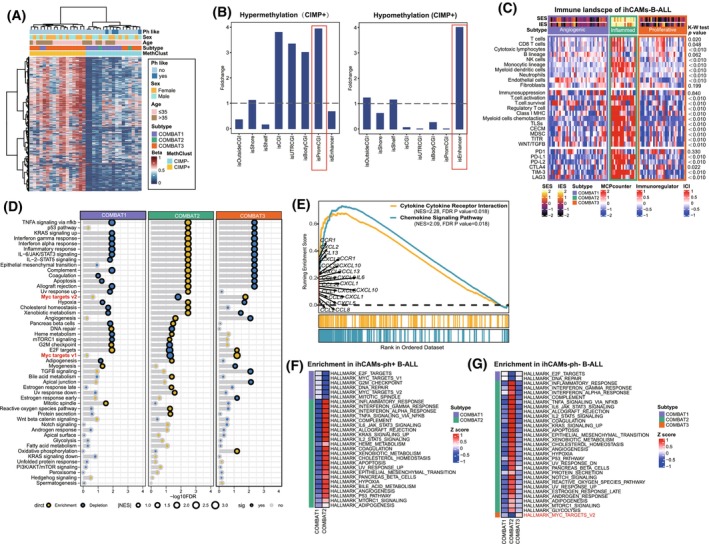
Differential demethylator phenotypes and immune landscape among three subtypes. (A) Hierarchical clustering of B‐ALL patients and healthy controls based on CpG sites with high methylation in normal controls (average beta‐value >0.9) and variable methylation in B‐ALL (beta‐value standard deviation >0.25). Two methylation‐based epi‐clusters: CIMP+ and CIMP‐ subgroups were identified. CIMP: CpG island methylator phenotype; CIMP+: hyper‐CIMP; CIMP‐: hypo‐CIMP. (B) Bar plot showing the region‐specific distribution of differentially methylated probes (DMPs) derived from the CIMP+ group in comparison to CIMP‐. In the CIMP+ group, hypermethylated probes were predominantly located in promoter regions(left), whereas hypomethylated probes were enriched in enhancer regions(right), as highlighted by the red box, respectively. CpG islands (CGIs) were annotated per UCSC Genome Browser, with flanking regions categorized as shore (0–2 kb), shelf (2–4 kb), or outside CGI (>4 kb). Sub‐annotations included promoter‐associated (isPromCGI; TSS ± 1 kb), UTR (isUTRCGI), and gene body CGIs (isBodyCGI). (C) Comparison of the immune landscape across the three COMBAT subtypes, including immune cell infiltration (ICI), key immune cell regulators, markers associated with immune exhaustion and immune checkpoint blockades, organized from top to bottom. Statistical significance was determined using Kruskal−Wallis (K–W) test. COMBAT1 was enriched with endothelial cells, defined as the angiogenic subtype. COMBAT2 displayed an inflamed subtype with increased numbers of T/NK and monocytic cells. COMBAT3 exhibited lower immune infiltration but significantly upregulated pathways and mutations associated with proliferation, defining it as proliferative. TITR, tumor‐infiltrating Tregs; MDSC, myeloid‐derived suppressor cells; TLS, tertiary lymphoid structures; C‐ECM, cancer‐associated extracellular matrix; SES, stromal enrichment score; IES, immune enrichment score. (D) The gene set enrichment analysis (GSEA) dot plot for hallmark pathways illustrating the changes of biological pathways involved among the three COMBAT subtypes. The blue dots indicate downregulated pathways, while the yellow dots indicate upregulated pathways. The size of the dots represents the |NES| (Normalized Enrichment Score) value, with larger dots corresponding to higher |NES| values. Statistical significance was determined using the Fisher exact test. The dots with black outlines indicate statistical significance, while those with gray outlines denote no statistical significance. (E) Enrichment of cytokine‐cytokine receptor interaction and chemokine signaling gene sets in the COMBAT2 group compared to the other subtypes. Statistical significance was determined using the Fisher exact test with Benjamini‐Hochberg correction. (F, G) The heatmaps show uniquely upregulated pathways in each of the three COMBAT subtypes for both Philadelphia chromosome (Ph)‐positive (F) and Ph‐negative (G) B‐ALL patients.

Considering the correlation between antigen expression and methylated CpG regions, we further explored the characteristics of the CIMP phenotype in 34 patients with Ph‐negative B‐ALL. Unsupervised hierarchical clustering of the most variable probes, compared with donor samples, revealed two methylation‐based epi‐clusters: hyper‐CIMP (CIMP+, *N* = 17) and hypo‐CIMP (CIMP−, *N* = 17) subgroups (Fig. 5A). We then assessed the associations between CIMP subgroups and the COMBAT classification. In the COMBAT3 group, 75.0% of the patients were categorized into the CIMP+ group, whereas 83.3% of the patients in the COMBAT1 group and 100% of the patients in the COMBAT2 group were classified into the CIMP‐ group. Notably, all patients (4/4) with a Ph‐like phenotype were distributed in the CIMP‐ group. In the CIMP+ group, hypermethylated probes were predominantly located in promoter regions, whereas hypomethylated probes were enriched in enhancer regions (Fig. 5B). These findings correlated with the significant probe‐gene pairs associated with the CIMP+ and CIMP‐ phenotypes, indicating that the presence of hypomethylation in enhancer regions in the CIMP+ subtype corresponds to high gene expression (Fig. S4A).

### 3.7 Delineation of the mutational spectra of COMBAT in the ihCAMs cohort

We further analyzed the genetic mutation profiles of the three COMBAT subtypes. In the ihCAMs cohort, B‐ALL patients generally presented with a low TMB across all three groups (Fig. S4B). Among the 10 most mutated genes, epigenetic regulators (*KMT2C* [72%] and *KMT2D* [54%]), chromatin remodeling genes (*ARID1B* [39%]), histone modifiers (*CREBBP* [25%] and *SETD2* [19%]), and DNA methylation genes (*TP53* [18%]) were the most frequent mutations among the three COMBAT subtypes (Fig. S4B). Additionally, we found that COMBAT3 had greater enrichment of *TP53* mutations (*P* = 0.002) in comparison to COMBAT1 (Fig. S4C).

When the overall mutation phenotype was considered, single‐base substitution (SBS) signature 1, which is related to transitions at methylated CpG sites, was significantly different among the three COMBAT subtypes (*P* = 0.030, Fig. S4B). This result corresponds to the above‐mentioned epigenetic‐related gene mutations and the epigenetic changes associated with antigen alterations.

### 3.8 Associations between distinct COMBAT immune profiles and biological pathways in B‐ALL patients

In view of the significant role of the immune microenvironment in the progression of ALL, we explored the immune landscape across the three subtypes by deconvoluted RNA‐seq data (Fig. 5C). COMBAT1 exhibited a low immune burden but a greater presence of endothelial cells, which are known to contribute hematopoietic cytokines to the perivascular bone marrow niche. COMBAT2 displayed an inflamed subtype with increased numbers of T/NK and monocytic cells. This group also presented increased expression of immune checkpoint genes, such as *PD‐L1*, *PD‐L2*, *CTLA4*, and *TIM‐3*, suggesting potential responsiveness to immunotherapy. Moreover, COMBAT2 exhibited an immunosuppressive environment with a higher stromal enrichment score (SES) compared with the other COMBAT groups, marked by high infiltration of cancer‐associated extracellular matrix (C‐ECM), tumor‐infiltrating T‐regulatory cells (TITRs), and myeloid‐derived suppressor cells (MDSCs). GSEA (Fig. 5D,E) linked COMBAT2 to immune response pathways, such as interferon and inflammatory responses, and revealed unique activation of cytokine–cytokine receptor interactions (NES 2.28, *P* = 0.018) and chemokine signaling pathways (NES 2.09, *P* = 0.018). Overall, COMBAT2 represents an inflamed yet immune‐exhausted subgroup.

In contrast, patients in COMBAT3 exhibited a low immune burden. However, a proliferative subtype was observed, which was characterized by significant upregulation of the *MYC* pathway as well as cell cycle pathways (G2M checkpoint and E2F targets, Fig. 5D). Furthermore, *KRAS/NRAS* mutations were highly prevalent in COMBAT3 (*NRAS P* = 0.0006, *KRAS P* = 0.0166), which further induced a hyper‐proliferative state (Fig. S4C).

Additionally, we performed enrichment analysis separately for Ph‐positive and Ph‐negative patients across different COMBAT subtypes and observed similar activated pathways in both groups (Fig. 5F,G). The distinct phenotypes identified by COMBAT suggested that B‐ALL patients with similar immune statuses may also share common biological processes.

### 3.9 Validation of COMBAT subtypes in external cohorts

**Fig. 6:**
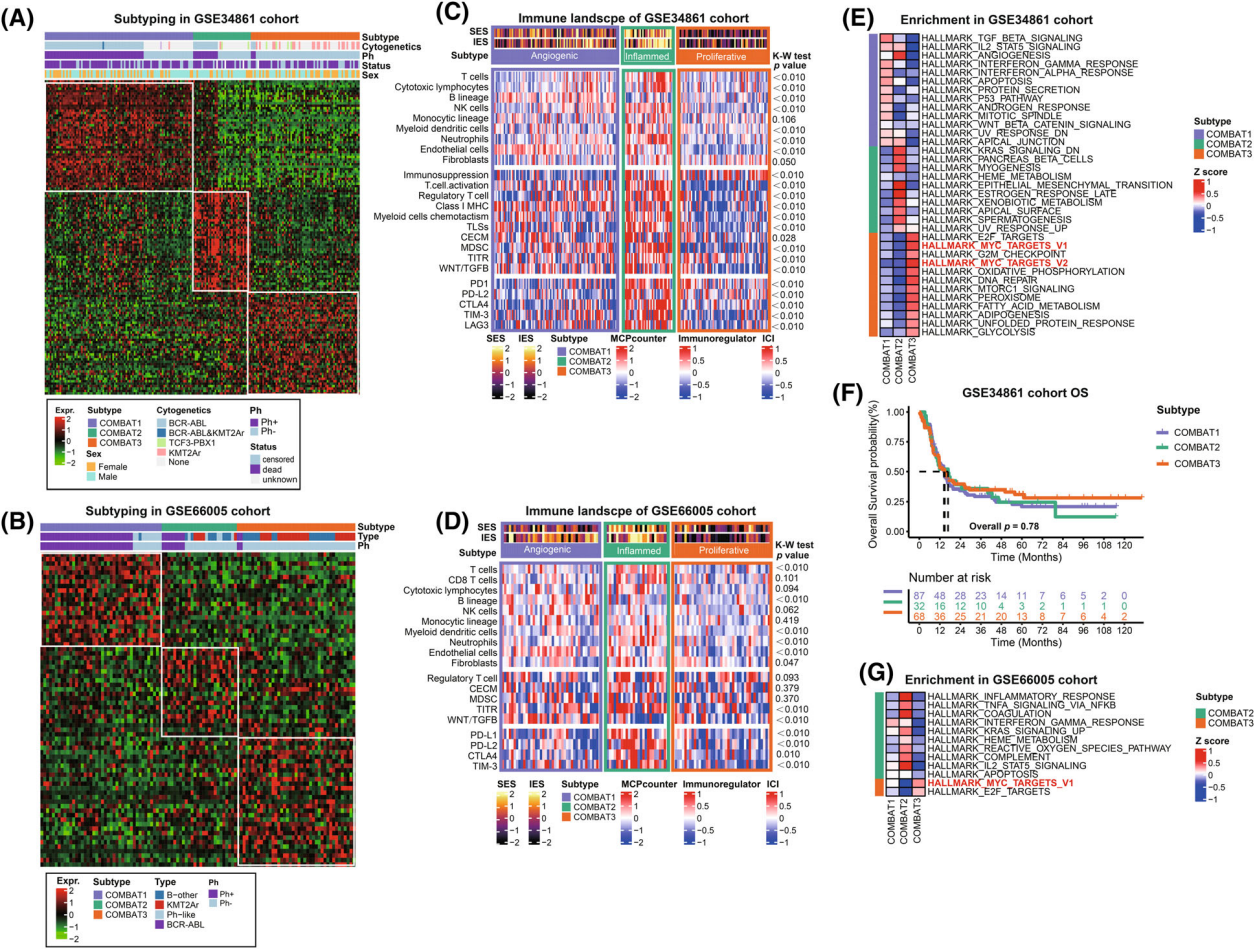
Successful validation of newly defined subtypes in two external cohorts. (A, B) Nearest template prediction (NTP) method was applied to predict the three subtypes for GSE34861 and GSE66005 cohort respectively by the subtype‐specific upregulated biomarkers in ihCAMs cohort. (C, D) Immune landscape of three subtypes in GSE34861 and GSE66005 cohort respectively, serving as a robust validation for the immunological characteristics inherent to COMBAT three subtypes. Statistical significance was determined using Kruskal−Wallis(K‐W) test. COMBAT1 was enriched with endothelial cells, defined as angiogenic subtype. COMBAT2 displayed an inflamed subtype with increased numbers of T/NK and monocytic cells. COMBAT3 exhibited lower immune infiltration but significantly upregulated pathways and mutations associated with proliferation, defining it as proliferative. TITR, tumor‐infiltrating Tregs; MDSC, myeloid‐derived suppressor cells; TLS, tertiary lymphoid structures; C‐ECM, cancer‐associated extracellular matrix; SES, stromal enrichment score; IES, immune enrichment score. (E) Heatmap shows the uniquely upregulated pathways of each subtype in GSE34861 cohort. (F) The Kaplan–Meier survival curve estimate of overall survival (OS) in the three subtypes in GSE34861 cohort. Statistical significance was determined using Fisher's exact test. (G) Heatmap shows the uniquely upregulated pathways of each subtype in GSE66005 cohort.

Considering the distinct transcriptomic landscapes observed across the three COMBAT subgroups, the top 50 upregulated unique biomarkers were identified for the three subtypes, with a significance threshold (adjusted *P* < 0.05). Using the 150‐gene signature (Table S2), we successfully replicated the COMBAT subtypes across two external cohorts, GSE34861 (*N* = 194) and GSE66005 (*N* = 109), as well as in 8 B‐ALL cell lines from the CCLE database, employing the NTP method (Fig. 6A,B; Fig. S5).

We further validated the characteristics of COMBAT and reported that Ph‐positive patients and a Ph‐positive cell line (SUP‐B15) were distributed mainly in COMBAT1‐2. COMBAT2 exhibited a high immune burden in both external cohorts. Dramatic activation of immune exhaustion signatures, such as TITR and WNT/TGFb, was also observed in the COMBAT2 group (Fig. 6C,D). With respect to signaling pathways, the *MYC* target pathway was upregulated in COMBAT3, which was consistent with the findings from the ihCAMs cohort (Fig. 6E,G). These comprehensive analyses across various datasets strongly supported the molecular, immune, and biological phenotypes observed in the COMBAT cohort. As for clinical outcomes, no significant differences in OS were observed among the three clusters in the GSE34861 cohort (Fig. 6F). Regrettably, survival data were not available in the GSE66005 cohort, and clinical information pertaining to HSCT was lacking in both external cohorts.

## 4 Discussion

In this study, B‐ALL exhibited unique omics profiles, revealing specific molecular pathways distinct from normal individuals. We subsequently conducted a comprehensive classification of adult B‐ALL utilizing COMBAT. Three COMBAT subtypes were identified through unsupervised clustering by multi‐omics integration, each with unique molecular patterns yet commonalities, especially in Ph‐negative patients.

COMBAT1 and COMBAT2 both showed significantly elevated expression of stem/myeloid‐associated surface antigens (*CD34*, *HLA‐DR*, *CD13*, and *CD33*) and genes (*GATA2*, *KIT*, *CSF3R*, etc.), which are linked to poor chemotherapy response and inferior survival in B‐ALL patients [34, 36, 37]. Accordingly, patients undergoing allo‐HSCT of COMBAT1 and COMBAT2 suffered poorer survival in our cohort.

In addition, COMBAT1 exhibited lower immune infiltration but greater enrichment of endothelial cells. Notably, leukemia cells can induce angiogenesis within the bone marrow microenvironment, and B‐ALL may rely on angiogenesis for its sustenance and progression [38]. On the contrary, COMBAT2 showed significantly higher ICI, upregulated markers of immunosuppression and immune exhaustion, and elevated ICB levels. These factors may contribute to immune tolerance, tumor escape [39, 40, 41, 42], and frequent disease recurrence during immunosuppressive treatment [43]. These observations may explain why COMBAT2 has a worse prognosis in comparison to COMBAT1.

In our study, the COMBAT3 subtype displayed significantly upregulated pathways and mutations associated with proliferation, such as *MYC* targets, cell cycle pathways, and *K/NRAS* mutations which are commonly linked to unfavorable outcomes. However, there was a notable improvement in the overall survival of COMBAT3 patients who underwent allo‐HSCT compared with COMBAT1‐2 patients. *ZNF384* rearrangement is a common genetic alteration in adult Ph‐negative B‐ALL, associated with improved survival in patients receiving allo‐HSCT [44]. A recent study found that patients with *KMT2A* rearrangements who underwent HSCT had a 5‐year overall survival rate of 32%, compared to 11% in those who did not receive HSCT [45]. These findings suggest that HSCT may offer a survival benefit for patients with unfavorable cytogenetic alterations. In our cohort, we observed that patients with *KMT2A* and *ZNF384* rearrangements were predominantly classified into the COMBAT3 group. Notably, a significant proportion of these patients (5/7, 71.4%) underwent allo‐HSCT. This may contribute substantially to the improved prognosis observed in the COMBAT3 group.

Aberrant DNA methylation is a characteristic feature of blood malignancies [46]. Borssén et al. [47] found that B‐ALL patients with a CIMP− profile at initial diagnosis had inferior overall survival compared with CIMP+ patients. The CIMP− group comprised more patients with unfavorable cytogenetic subtypes [47]. In our study, while there was no significant difference in survival between the CIMP+ and CIMP− groups (OS, *P* = 0.854), unfavorable Ph‐like patients were enriched in the CIMP− group. Furthermore, patients in the COMBAT1‐2 groups, which were associated with poorer prognosis, were predominantly in the CIMP− group, whereas the majority of COMBAT3 patients were in the CIMP+ group. Although a larger cohort is needed in future studies, these findings suggest that our classification of B‐ALL holds promise in identifying the CIMP profile.

This study presents several key findings and advantages. First, we employed 10 clustering algorithms, integrated via consensus ensembles, ensuring a more robust classification system compared to studies relying on a single method. Second, beyond NCCN recommendations [35], emerging evidence underscores the critical roles of the immune microenvironment, myeloid‐related gene expression, and epigenetics in shaping B‐ALL outcomes [34, 36, 37, 47, 48, 49]. The COMBAT classification, incorporating multidimensional factors, delineates B‐ALL's biological heterogeneity and enhances survival outcome predictions across subtypes.

Third, the COMBAT classification captures distinct transcriptomic landscapes that define key biological processes in B‐ALL. COMBAT1 is marked by endothelial remodeling, with increased expression of endothelial‐associated genes, suggesting vascular niche alterations that may influence leukemic cell survival and therapy response. Given prior evidence linking non‐classical monocytes to vascular damage in pediatric B‐ALL [50], similar immune–vascular interactions may contribute to endothelial remodeling in COMBAT1. However, due to significant biological differences between adult and pediatric B‐ALL, direct comparisons remain challenging. Future single‐cell RNA sequencing could help delineate monocyte subsets and their role in shaping the vascular niche, providing deeper insights into B‐ALL pathogenesis. The endothelial involvement in COMBAT1 also suggests that anti‐angiogenic therapies may be a viable therapeutic strategy. Additionally, COMBAT2 exhibits features of immune exhaustion, with elevated expression of immune checkpoint genes (e.g., PD‐L1, CTLA4) and T‐cell dysfunction markers, highlighting potential vulnerabilities for immune checkpoint blockade therapies. Meanwhile, COMBAT3 is MYC‐driven, characterized by enriched MYC‐related pathways and proliferative gene signatures, which may contribute to its aggressive phenotype and poor prognosis, underscoring the need for targeted therapies against MYC signaling. These mechanistic insights provide a molecular basis for therapeutic stratification, offering a refined framework for subclassifying B‐ALL and guiding treatment strategies. By integrating these biological features, the COMBAT classification not only enhances our understanding of B‐ALL heterogeneity but also lays the foundation for future precision medicine approaches in leukemia.

Fourth, given the dismal prognosis of Ph‐negative B‐ALL, our study primarily focuses on uncovering the genetic, transcriptomic, and methylated landscape of Ph‐negative B‐ALL. We also explore potential transplantation benefits for the Ph‐negative patient subgroup, underscoring the clinical relevance of our findings.

Fifth, leveraging the NTP method, we identified a 150‐gene signature that enables COMBAT subtype classification solely based on RNA‐Seq data, making it a practical and scalable approach given the widespread use of bulk RNA sequencing. Finally, as our classifier was trained on patients treated with intensive chemotherapy, it is particularly suited for guiding treatment decisions in cases where immunotherapy is not a frontline option. Notably, for COMBAT2 patients, characterized by an inflamed and immune‐exhausted profile, immunotherapy may represent a promising alternative.

The limitations of this study include the small sample size of the ihCAMs‐B‐ALL cohort and the limited availability of adult B‐ALL RNA‐seq data in public datasets. Survival data were not available for the GSE66005 cohort, and clinical information pertaining to HSCT was lacking in both external cohorts. The absence of HSCT data constrained our ability to fully assess its impact on genetic stratification and survival outcomes. However, the molecular characteristics of each COMBAT subtype were confirmed in the validation cohort. Future studies should address these limitations by enrolling larger patient cohorts and incorporating comprehensive transplantation data to better understand the interplay between genetic factors and clinical outcomes. Additionally, the absence of proteomic and metabolomic data in our study limits our ability to capture post‐translational modifications, protein interactions, and metabolic reprogramming—key factors necessary for a comprehensive understanding of B‐ALL biology. Nevertheless, by integrating genomic, transcriptomic, and methylome features, our study represents one of the most comprehensive multi‐omics classifications of B‐ALL to date.

## 5 Conclusion

Overall, our study presents a comprehensive omics‐based classification for adult B‐ALL patients. This classification not only predicts immune characteristics but also delineates the potential prediction of benefits from allo‐HSCT. These findings provide crucial insights into therapeutic options and outcomes for adult B‐ALL patients, thereby enhancing our understanding of effective treatment strategies.

## Conflict of interest

The authors declare no competing interests.

## Author contributions

YS performed bioinformatics and functional analysis. TL designed the research and wrote the manuscript. YS and TL contributed equally as co‐first authors. QH and QF collected the clinical samples. XG, YL, and ZT provided patient samples and integrated the clinical data. MW, HW, and JW assisted in data interpretation. TC and YM designed and supervised the research.

## Supporting information


**Fig. S1.** Principal component analysis (PCA) of all patients in ihCAMs‐B‐ALL cohort, categorized by COMBAT.
**Fig. S2.** Survival outcomes of all patients across the three subtypes in ihCAMs‐B‐ALL cohort.
**Fig. S3.** Survival outcomes of Philadelphia chromosome (Ph)‐positive patients in ihCAMs‐B‐ALL cohort.
**Fig. S4.** Genetic mutation profiles of three subtypes in ihCAMs‐B‐ALL cohort.
**Fig. S5.** Validation of COMBAT subtypes across CCLE B‐ALL cell lines employing the nearest template prediction (NTP) method.
**Table S1.** Targeted exome sequencing 267 gene panel.
**Table S2.** Subtype‐specific genes for COMBAT.

## Data Availability

The raw sequence data were available in the Genome Sequence Archive in the National Genomics Data Center built by the China National Center for Bioinformation/Beijing Institute of Genomics, Chinese Academy of Sciences with accession number PRJCA023183 (HRA006693 and OMIX005777).
