# Supplementary material for: Comprehensive omics‐based classification system in adult patients with B‐cell acute lymphoblastic leukemia
Source: Mol Oncol. 2025 May 19;19(12):3578–95. doi: 10.1002/1878-0261.70053 (PMC12688170; doi:10.1002/1878-0261.70053)
Supplement: Supplementary file 1 — Fig. S1. Principal component analysis (PCA) of all patients in ihCAMs‐B‐ALL cohort, categorized by COMBAT. Fig. S2. Survival outcomes of all patients across the three subtypes in ihCAMs‐B‐ALL cohort. Fig. S3. Survival outcomes of Philadelphia chromosome (Ph)‐positive patients in ihCAMs‐B‐ALL cohort. Fig. S4. Genetic mutation profiles of three subtypes in ihCAMs‐B‐ALL cohort. Fig. S5. Validation of COMBAT subtypes across CCLE B‐ALL cell lines employing the nearest template prediction (NTP) method. Table S1. Targeted exome sequencing 267 gene panel. Table S2. Subtype‐specific genes for COMBAT. [file MOL2-19-3578-s001.docx]

**Comprehensive omics-based classification system in adult patients with B-cell acute lymphoblastic leukemia**

**Running title：** Multi-omics classification of B-ALL

**Authors and affiliations**: Yang Song^1,2*^, Ting Liu^1,2*^, Qishan Hao^1^, Qiuyun Fang^1,2^, Xiaoyuan Gong^1,2^, Yan Li^1,2^, Zheng Tian^1,2^, Hui Wei^1,2^, Min Wang^1,2^, Jianxiang Wang^1,2^, Tao Cheng^1,2#^, Yingchang Mi^1,2#^

1. *State Key Laboratory of Experimental Hematology, National Clinical Research Center for Blood Diseases, Haihe Laboratory of Cell Ecosystem, Institute of Hematology & Blood Diseases Hospital, Chinese Academy of Medical Sciences & Peking Union Medical College.*
2. *Tianjin Institutes of Health Science, Tianjin China*

* Yang Song and Ting Liu contributed equally to this study.

**#Corresponding author:**

**Yingchang Mi, MD**

State Key Laboratory of Experimental Hematology, National Clinical Research Center for Blood Diseases, Haihe Laboratory of Cell Ecosystem, Institute of Hematology & Blood Diseases Hospital, Chinese Academy of Medical Sciences & Peking Union Medical College, 288 Nanjing Road, Tianjin 300020, China.

E-mail address: ychmi@ihcams.ac.cn

**Tao Cheng, MD ＆ PhD**

State Key Laboratory of Experimental Hematology, National Clinical Research Center for Blood Diseases, Haihe Laboratory of Cell Ecosystem, Institute of Hematology & Blood Diseases Hospital, Chinese Academy of Medical Sciences & Peking Union Medical College, 288 Nanjing Road, Tianjin 300020, China.

E-mail address: chengtao@ihcams.ac.cn

**Supplementary Table**

**Supplementary Table 1: Targeted exome sequencing 267 gene panel**

| ABCB1 | BRIP1 | CREBBP | EPOR | HAX1 | KLHL6 | NFKBIE | PRF1 | SF3B1 | TMEM30A |
| --- | --- | --- | --- | --- | --- | --- | --- | --- | --- |
| ABL1 | BTG1 | CRLF2 | ETNK1 | HLA-A | KMT2A | NOTCH1 | PRKCB | SGK1 | TMSBX |
| ANKRD26 | BTG2 | CSF1R | ETV6 | HLAC | KMT2B | NOTCH2 | PRKD2 | SH2B3 | TNFAIP3 |
| APC | BTK | CSF3R | EZH2 | HLA-DMB | KMT2C | NOTCH3 | PRKDC | SH2D1A | TNFRSF14 |
| ARID1A | CALR | CSMD1 | FAM46C | HNRNPK | KMT2D | NOTCH4 | PRPF8 | SMARCA4 | TOX |
| ARID1B | CARD11 | CSNK1A1 | FAS | HRAS | KRAS | NPM1 | PRPS1 | SMC1A | TP53 |
| ARID2 | CBL | CTCF | FAT1 | HUWE1 | KRT20 | NRAS | PSMB5 | SMC3 | TPMT |
| ARID5B | CBLB | CUX1 | FAT4 | HVCN1 | LCOR | NT5C2 | PTEN | SMO | TRAF3 |
| ASXL1 | CBLC | CXCR4 | FBXO11 | ID3 | LM02 | P2RY8 | PTPN1 | SOCS1 | U2AF1 |
| ASXL2 | CCND1 | CYLD | FGFR3 | IDH1 | LTB | PALB2 | PTPN11 | SP140 | UBE2A |
| ATG2B | CCND3 | DDX3X | FLT3 | IDH2 | LYN | PAX5 | PTPRD | SPEN | UBR5 |
| ATM | CCR4 | DDX41 | FOXO1 | IGLL5 | MAP2K1 | PBXW7 | RAD21 | SRP72 | USP7 |
| ATRX | CD28 | DIS3 | FYN | IKZF1 | MAPK1 | PDGFRA | RASA2 | SRSF2 | VAV1 |
| B2M | CD58 | DKC1 | GAB2 | IKZF2 | MAX | PDGFRB | RB1 | STAG2 | VHL |
| BACH2 | CD79A | DNM2 | GATA1 | IKZF3 | MCL1 | PDS5B | RBBP6 | STAT3 | WHSC1 |
| BCL10 | CD79B | DNMT3A | GATA2 | IL7R | MED12 | PHF6 | RELN | STAT5B | WT1 |
| BCL2 | CDC25C | DNMT3B | GATA3 | IRF2BP2 | MEF2B | PIGA | RHOA | STAT6 | XBP1 |
| BCL6 | CDKN1A | DTX1 | GFI1 | IRF4 | MFHAS1 | PIK3CA | RPL10 | SUFU | XPO1 |
| BCL7A | CDKN1B | DUSP2 | GNA13 | IRF8 | MPL | PIK3CD | RRAGC | SUZ12 | ZAP70 |
| BCOR | CDKN2A | EBF1 | GNAI2 | ITPKB | MTOR | PIK3R1 | RUNX1 | SYK | ZBTB7A |
| BCORL1 | CDKN2B | EED | GNAS | JAK1 | MYC | PIM1 | SAMHD1 | TAL1 | ZFP36L1 |
| BIRC3 | CDKN2C | EGFR | GNB1 | JAK2 | MYCN | PIM2 | SBDS | TBL1XR1 | ZMYM3 |
| BLM | CEBPA | EGLN1 | GSKIP | JAK3 | MYD88 | PLCG1 | SETBP1 | TCF3 | ZNF292 |
| BPGM | CHD2 | EGR1 | H1-2 | JUNB | MYOM2 | PLCG2 | SETD1B | TERC | ZRSR2 |
| BRAF | CHD8 | ELANE | H1-3 | KDM6A | NF1 | POT1 | SETD2 | TERT |  |
| BRCA1 | CIITA | EP300 | H1-4 | KIT | NFE2 | PPM1D | SETDB1 | TET1 |  |
| BRCA2 | CNOT3 | EPHA7 | H1-5 | KLF2 | NFKBIA | PRDM1 | SF1 | TET2 |  |

**Supplementary Table 2: Subtype specific genes for COMBAT**

| **COMBAT1-specific** | **COMBAT2-specific** | **COMBAT3-specific** |
| --- | --- | --- |
| CCL17 | CCL2 | CSMD1 |
| PRX | CCL8 | PCDH9 |
| DPEP1 | CXCL5 | RGS1 |
| CTHRC1 | CXCL1 | SALL4 |
| WNT9A | CD14 | KCNQ5 |
| ECM1 | CCL7 | BLK |
| BAALC | SERPINB2 | AEBP1 |
| TSPAN7 | PLA2G7 | VANGL2 |
| S100A16 | ADAMDEC1 | VLDLR |
| CD34 | PI3 | NXN |
| ARHGEF17 | CXCL10 | GNAZ |
| CYTL1 | CYP27A1 | RGS16 |
| NRXN3 | CCL22 | ZNF711 |
| SOCS2 | CHI3L1 | APBA1 |
| MME | CTSL | SPINK2 |
| EFNA1 | TNFAIP6 | PLXNB1 |
| C1QTNF4 | CYP1B1 | IGLL1 |
| MUC4 | IL6 | NFATC4 |
| S100Z | FCER1G | GPM6B |
| SLC45A3 | C3 | CAPSL |
| OLFML2A | CXCL3 | SNX22 |
| BMPR1B | FFAR2 | RGS2 |
| P2RY14 | CCL13 | CAMK2D |
| MYO1B | IL1RN | VPREB1 |
| PON2 | CXCL2 | SLC16A9 |
| NPDC1 | TMEM176B | PKIA |
| CAPN11 | PTAFR | LDLRAD3 |
| CYGB | AQP9 | IRS1 |
| CMTM2 | MAFB | IRX1 |
| DUSP26 | HMOX1 | WASF1 |
| EGFL7 | SIGLEC1 | P2RX5 |
| KAZALD1 | TLR8 | CD38 |
| MMRN1 | CLEC7A | BCAS4 |
| FGF6 | SEMA6B | PRL |
| CD99 | FPR1 | ADPRHL1 |
| FLT4 | CD163 | RASAL1 |
| SEMA6A | IER3 | ESAM |
| PTP4A3 | IL4I1 | APBB2 |
| POU4F1 | EBI3 | PPM1H |
| ROBO4 | FCGR1A | NR4A1 |
| EMP1 | LAMP3 | KCNMB4 |
| CA6 | ANKRD22 | SNX25 |
| BMP2 | ADM | SHROOM3 |
| EPAS1 | CCR1 | MGAT5B |
| SLC2A5 | S100A11 | ADD2 |
| BCL6B | VSIG4 | PHACTR3 |
| RAG1 | SLAMF7 | RAG2 |
| STK32B | LRRC25 | FMNL2 |
| FBXW7 | GPR84 | BACH2 |
| NPR1 | SERPINA1 | GPT2 |

**Supplementary Figure**

**Figure S1**

**
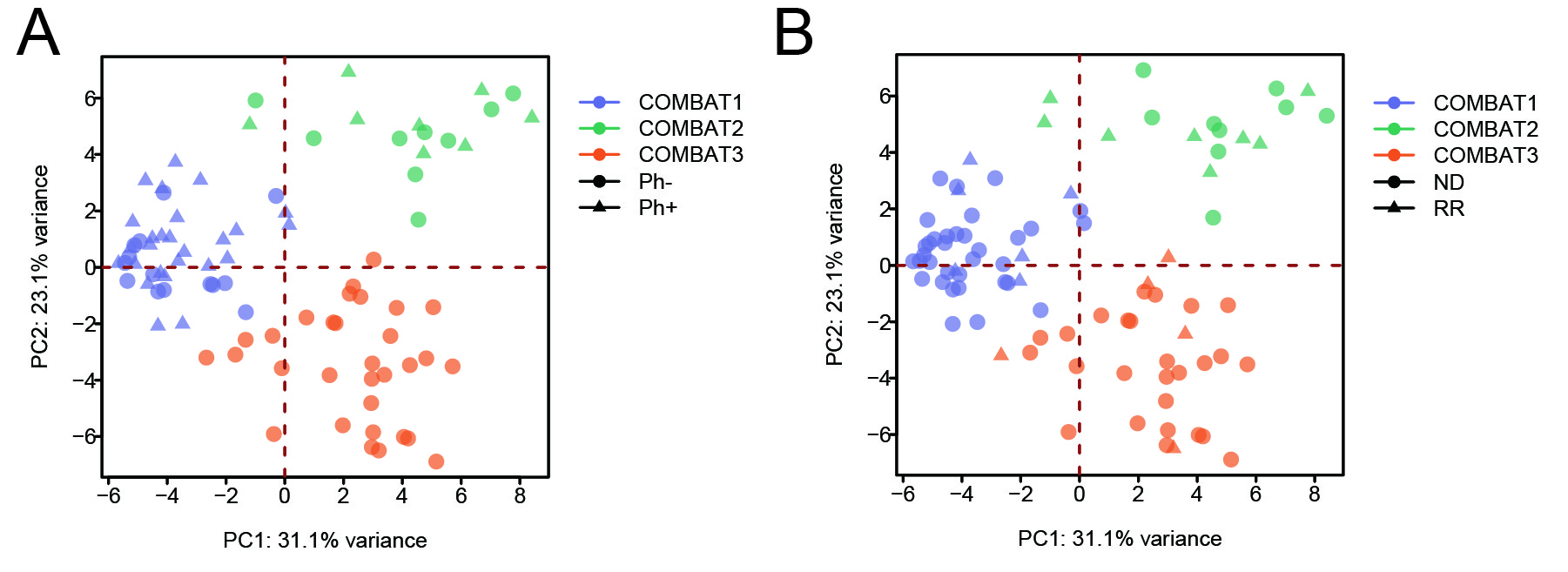
**

**Figure S1. Principal component analysis (PCA) of all patients in ihCAMs-B-ALL cohort**, **categorized by COMBAT.**

**A.** PCA plot of Philadelphia chromosome-negative (Ph-) patients (circles) and Philadelphia chromosome-positive (Ph+) patients (triangles) based on the 150 most variable genes (listed in Supplementary Table 2) under COMBAT classification.

**B.** PCA plot of newly-diagnosed (ND) patients (circles) and refractory/relapsed (RR) patients (triangles) based on the 150 most variable genes (listed in Supplementary Table 2) under COMBAT classification.

**Figure S2**


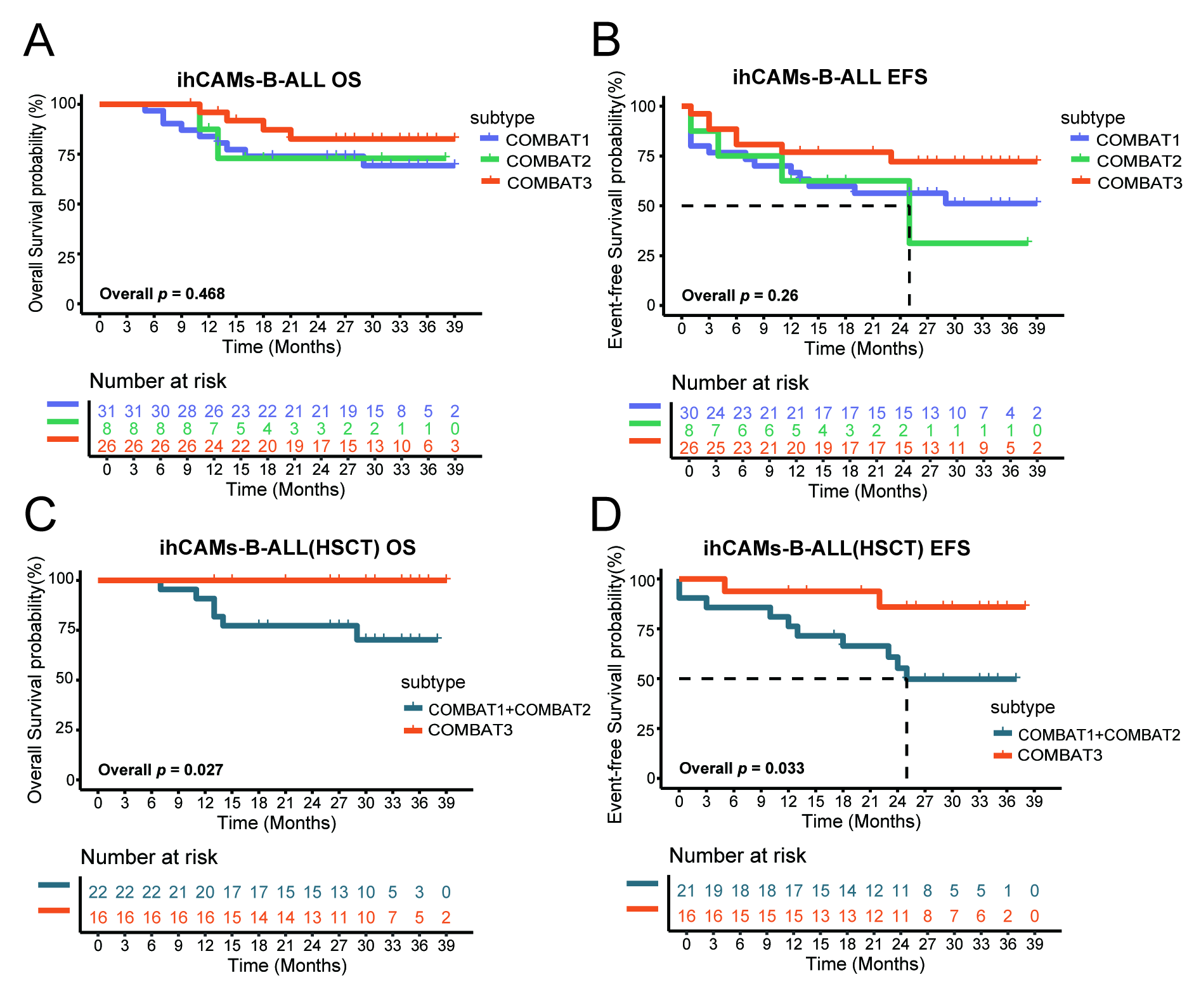


**Figure S2. Survival outcomes of all patients across the three subtypes in ihCAMs-B-ALL cohort.**

**A-B.** The Kaplan–Meier survival curve compares the overall survival (OS, **A**) and event-free survival (EFS, **B**) differences across the three COMBAT subtypes. Statistical significance was determined using Fisher’s exact test.

**C-D.** The Kaplan–Meier survival curves compare overall survival (OS, **C**) and event-free survival (EFS, **D**) in COMBAT1/2 vs. COMBAT3 patients who undergoing allogeneic hematopoietic stem cell transplantation (allo-HSCT), respectively. Statistical significance was determined using Fisher’s exact test.

**Figure S3**

**
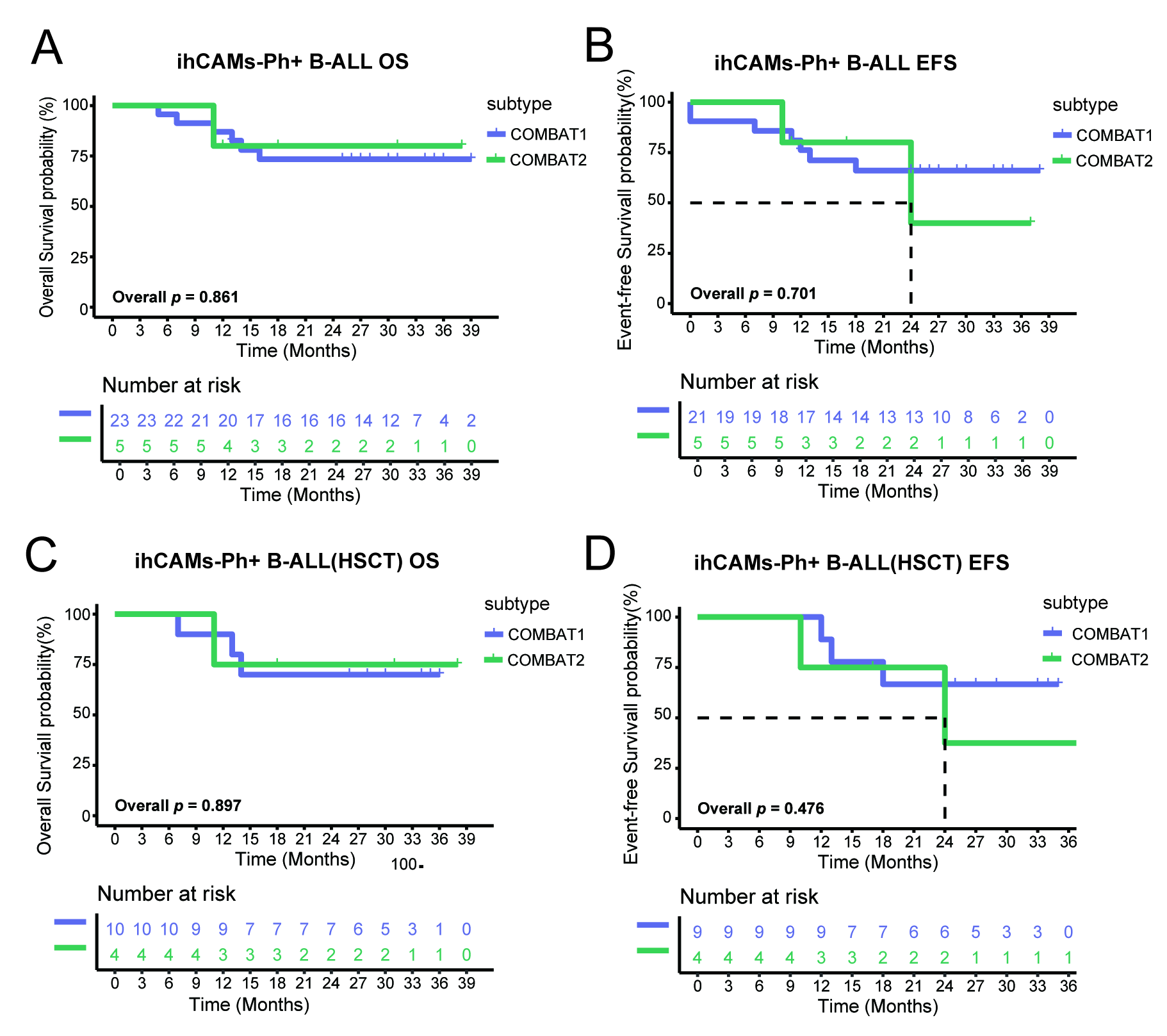
**

**Figure S3. Survival outcomes of Philadelphia chromosome (Ph)-positive patients in ihCAMs-B-ALL cohort.**

**A-B.** The Kaplan–Meier survival curves illustrate the differences in overall survival (OS, **A**) and event-free survival (EFS, **B**) between the two subtypes of Philadelphia chromosome (Ph)-positive patients (COMBAT1 vs. COMBAT2), respectively. Statistical significance was determined using Fisher’s exact test.

**C-D.** The Kaplan–Meier survival curve compares the overall survival (OS, **D**) and event-free survival (EFS, **D**) differences across the two subtypes of Ph-positive patients undergoing allogeneic hematopoietic stem cell transplantation (allo-HSCT, COMBAT1 vs. COMBAT2), respectively. Statistical significance was determined using Fisher’s exact test.

**Figure S4**

**
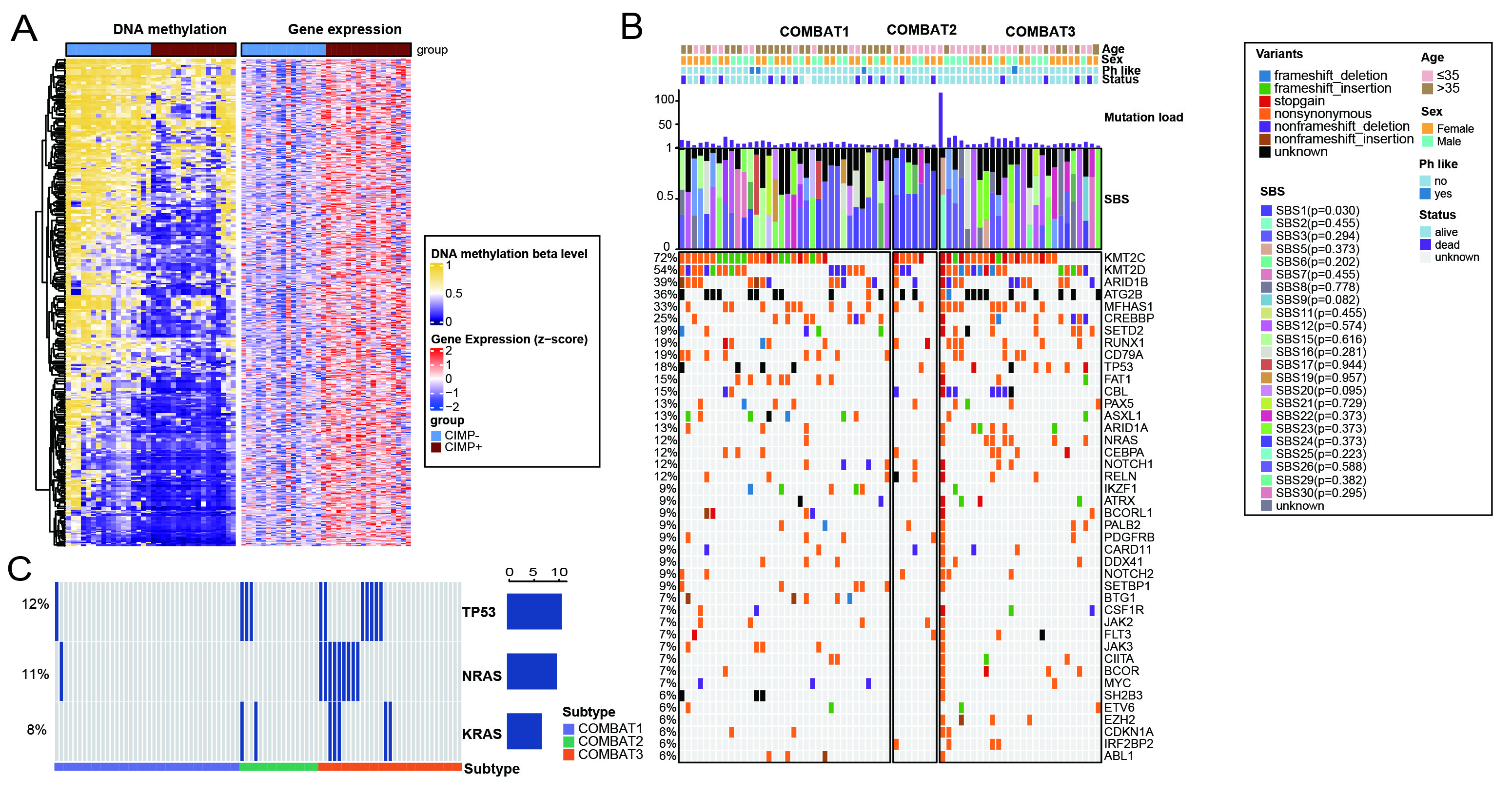
**

**Figure S4. Genetic mutation profiles of three subtypes in ihCAMs-B-ALL cohort.**

**A.** Combined analysis of significant probe-gene pairs in CIMP+ and CIMP- phenotypes. Note: CIMP: CpG island methylator phenotype; CIMP+：hyper-CIMP; CIMP-: hypo-CIMP.

**B.** Tumor mutational burden (TMB), single-base substitution (SBS) signature and genetic mutation distribution of three COMBATs in ihCAMs-B-ALL cohort, organized from top to bottom. TMB expressed in mutations /Mb, frequency of commonly mutated genes, and the alteration ratio of the genome.

**C.** The most differential gene mutations identified by the comparison across the three COMBAT subtypes.

**Figure S5**

**
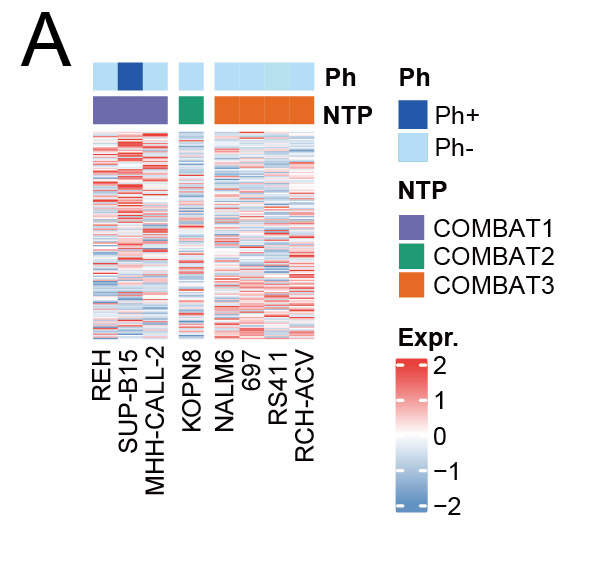
**

**Figure S5. Validation of COMBAT subtypes across CCLE B-ALL cell lines employing the nearest template prediction (NTP) method.**

A Heatmap of 8 B-ALL cell lines derived from CCLE database, including 1 Philadelphia chromosome (Ph)-positive and 7 Ph-negative cell lines, subtyped by COMBAT.
